# Reduction of Dental Caries Among Children and Adolescents From a 15-Year Community Water Fluoridation Program in a Township Area, Korea

**DOI:** 10.3390/ijerph16071306

**Published:** 2019-04-11

**Authors:** Han-Na Kim, Wook-Sung Kong, Jung-Ha Lee, Jin-Bom Kim

**Affiliations:** 1 Department of Dental Hygiene, College of Health and Medical Sciences, Cheongju University, Cheongju 28503, Korea; hnkim@cju.ac.kr; 2 Department of Preventive and Community Dentistry, School of Dentistry, Pusan National University, Yangsan 50612, Korea; ss0016@hanmail.net (W.-S.K.); Jhlee86@pusan.ac.kr (J.-H.L.); 3 BK PLUS Project, School of Dentistry, Pusan National University, Busan 50612, Korea

**Keywords:** caries prevention, dental caries, DMFT, fluoride, permanent teeth, water fluoridation

## Abstract

Since 2000, a community water fluoridation program (CWFP) has been implemented in Hapcheon for over 15 years. We aimed to evaluate the caries-reducing effect on permanent teeth after this implementation. In 2015, evaluation surveys were conducted by our study group, 498 subjects aged 8, 10, 12, and 15 years. As the control, 952 similarly aged subjects were selected from the Sixth Korean National Health and Nutrition Examination Survey (2013-2015 KNHANES). Data of a prospective cohort of 671 8-,10- and 12-year-olds, collected when CWFP started, were used for the evaluation. Caries-reducing effects were estimated by decayed, missing and filled teeth (DMFT) scores between CWFP and control groups, pre- and post-program. Confounders including the mean number of sealant teeth and gender were adjusted for. The mean adjusted DMFT scores of 10-, 12- and 15-year-olds in Hapcheon were significantly lower compared to KNHANES DMFT scores; in addition, those of 8-, 10- and 12-year-olds after the 15-year CWFP were significantly lower than in 2000. The caries-reducing effect among 12-year-olds was 37.6% compared to those recorded in KNHANES, and 67.4% compared to those in 2000. In conclusion, the caries-reducing effect was so high that health policy makers should consider CWFP as a priority policy for caries-reducing in Korean children and adolescents.

## 1. Introduction

In 2017, among the top ten major diseases that required out-patient expenses to be covered by the National Health Insurance (NHI), Korea, the conditions classified as ‘other disorders of the teeth and supporting structure’ had the highest expenses with costs of 1174 million USD; further, the expenses for ‘pulpitis and root apical lesion’ were in the 6th place with costs of 535 million USD, followed by those for ‘dental caries,’ in the 10th place, with costs of 294 million USD [1]. ‘Other disorders of the teeth and supporting structures’ is a diagnostic phrase that is widely applied to conditions requiring treatments involving dentures and implants; it is broadly defined as tooth loss that occurs owing to periodontal diseases, dental caries, and diseases that cannot be cured adequately and that progress severely [2]. Conditions categorized as ‘pulpitis and root apical lesion’ are also mostly caused by progressive dental caries. Therefore, dental caries is a pandemic disease that is experienced irrespective of the area a patient lives in and their age; further, it poses a threat to the public oral health and is an economic burden. The prevalence of dental caries among children and adolescents in Korea has decreased compared to in the past [3]. However, the prevalence of dental caries among 12-year-old children and 15-year-old adolescents still surpasses 50% [4]. 

In recent years, the most effective method of reducing dental caries has been the use of sealants and fluoride [5,6]. The community water fluoridation program (CWFP) is the first developed public health intervention that has been considered to reduce the prevalence of dental caries among a community’s residents by using fluoride [7]. The CWFP involves the addition of a controlled amount of fluoride at a constant concentration of 0.7 to 1.2 part per million (ppm) to the public water supply with the intent of reducing dental caries among the population [8]. 

The World Health Organization (WHO) adopted the “Oral health within WHO strategic directions 53.17: Prevention and control of non-communicable diseases” at its 53rd General Assembly in 2000. The WHO has stated that: “Community water fluoridation is effective in reducing dental caries in both children and adults. Water fluoridation benefits all residents served by community water supplies regardless of their social or economic status” [9]. In Korea, the first CWFP was initiated in 1981 in Jinhae City. It was later expanded to include 32 local areas and 36 water treatment plants to cover 8.9% of the Korean population in 2002 [10]. However, in recent times, the misunderstandings of local residents in relation to the effects of the CWFP and the passive attitude of civil servants have reduced the number of CWFP implementations. In 2016, the CWFP was implemented in only 13 counties [11]. In Korea, implementing the CWFP is included in the basic plan of the public oral health program in accordance with the Oral Health Law, and the head of the local government is responsible for establishing, implementing, and managing detailed enforcement plans in order to set up the CWFP [12]. 

In Grand Rapids (Michigan, USA); New Haven (Illinois, USA); Evanston (Illinois, USA); and Brantford (Ontario, Canada), where the CWFP was first introduced in 1945, the mean number of decayed, missing and filled teeth (DMFT scores), measured after the program was implemented for 13–14 years and compared with the scores at the beginning of the project or with those of a city without the program, decreased from 48.4% to 70.1% [13]. There have been many reports from around the world regarding caries reduction owing to the CWFP [14]. In Korea, Kim et al. [15] reported that the caries-reducing effect on permanent dentition due to the CWFP in Cheongju city was 22.2% to 46.2% among 7- to 11-year-old children. 

The DMFT score is one of the simplest and most commonly used indices in epidemiological surveys conducted on dental caries. It quantifies the dental health status based on the number of decayed, missing, and filled permanent teeth. DMFT scores have been used for more than 50 years and are well established as key measures of the prevalence of caries in dental epidemiological studies [16].

Since 1996, the Hapcheon Public Health Center started a sealant program that involved the first molar of primary school children in order to reduce the prevalence dental caries among local residents [17]. Since then, the target of children being administered public dental health care has gradually been expanded. Since 2006 onwards, a dental sealant program was implemented in primary schools that involved the sound pre-molars and molars of children from the 5th and 6th grades. On the other hand, since 2000, the CWFP has been mainly implemented in the Hapcheon township area [18]. Although there have been many reports on the reduction of caries in urban areas under the CWFP, there have been few studies on the effects of a prolonged CWFP implementation on dental caries in a suburban township area.

When communities have not implemented any caries-reducing program, a higher rate of prevalence of dental caries and higher DMFT scores are reported in suburban rural areas compared to in urban areas [19]. There is a need to continue research on the effects of the CWFP on dental caries reduction in suburban rural areas. Per a survey conducted in 2015, only four of the suburban areas in Korea had implemented the CWFP [11]. Meyer et al. [20] reported that CWF cessation promoted a marked increase in the number of caries-related procedures and treatment costs for Medicaid-eligible children and adolescents aged 0–18 years.

Since the time the CWFP began in the Hapcheon township area, it has been a place where the fluoride level in tap water has been stable and well maintained in order to reduce the incidence of dental caries. Therefore, it was selected as a study area. The hypothesis is as follows: (1) The mean DMFT adjusted for the number of sealant teeth and gender of sample was not significantly different between CWFP area and nationwide control in 2015 when the CWFP had been implemented for 15 years. (2) The mean DMFT adjusted for the number of sealant teeth and gender of sample was not significantly different between the year of 2000 and 2015 due to the 15-year implementation of CWFP in Hapcheon township area. Thus, the authors analyzed the caries-reducing effect of a suburban CWFP implementation, through the span of 15 years, on the local children and adolescents from the period between 2000 to 2015.

## 2. Materials and Methods

### 2.1. Participants

When the CWFP started in the Hapcheon township area in 2000, all the 8-, 10- and 12-year-old children, attending two primary and two middle schools in Hapcheon, were assigned to the CWFP group and were surveyed for oral examinations. The subjects of the oral examinations were 671 children: 213 of them were aged 8 years; 198, 10 years; and 260, 12 years [17]. The control group included 3603 children among the participants in the Korean National Oral Health Survey conducted by the Ministry of Health and Welfare in 2000; their ages were as follows: 1194 of them were aged 8 years; 1206, 10 years; and 1203, 12 years [21].

In 2015, 15 years after the introduction of the CWFP, all of the 8-, 10-, 12-, and 15-year-old children and adolescents in Hapcheon were selected as the study group for the evaluation of the caries-reducing effect derived from the CWFP implementation. They were all children and adolescents attending two primary, two middle, and two high schools. The children and adolescents who actually participated in the oral examinations were same ones who attended the respective schools on the day the oral examiner visited them from the period between June to July 2015. Thus, the number of total surveyed participants was 498 children and adolescents (103 aged 8, 116 aged 10, 117 aged 12, and 162 aged 16). 

**Figure 1:**
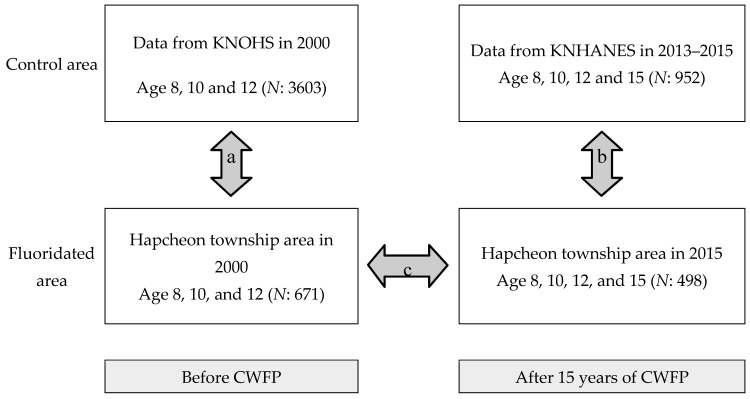
Participants and study procedure for the evaluation of the caries-reducing effect derived from the CWFP on permanent dentition. Note: a. The DMFT scores from the data from the Korean National Oral Health Survey conducted in 2000 and the DMFT scores from Hapcheon recorded in 2000 were adjusted for confounding factors including the mean number of sealant teeth and gender. Consequently, there were no significant differences between the adjusted DMFT scores of the two regions in 2000; b. Comparison between the data of the 2013–2015 control group and 2015 Hapcheon group using the DMFT scores and the estimation of the reduction effect; c. Estimation of caries-reducing effect on permanent dentition following the 15-year program by comparing adjusted DMFT scores from 2000 and 2015 in Hapcheon. CWFP: community water fluoridation program.KNHANES: Korea National Health and Nutrition Examination Survey. KNOHS: Korean National Oral Health Survey.

The control group consisted of 952 children and adolescents in total (243 aged 8, 239 aged 10, 239 aged 12, and 231 aged 15), who participated in oral examinations of the Sixth Korea National Health and Nutrition Examination Survey (KNHANES) conducted by the Korea Centers for Disease Control and Prevention (KCDC) (Table 1, Figure 1). All subjects gave their informed consent for inclusion before they participated in the study. The study was conducted in accordance with the Declaration of Helsinki, and the ethical approval for the study was obtained from the Pusan National University Dental Hospital Institutional Review Board (PNUDH-2015-013).

### 2.2. Oral Examination

A dentist surveyed the prevalence of dental caries and sealant teeth in children and adolescents in the Hapcheon township area in 2000 and 2015. He received training courses on inter-examiner reliability, participated in the Korean National Oral Health Survey in 2000, and received a training course on investigator guidance from the KNHANES for the 4th (2007–2009), 5th (2009–2012), and 6th (2013–2015) surveys conducted by the KCDC. In accordance with the WHO criteria for oral health surveys [22], the oral examinations on the participants were conducted under a blue-white portable examination light (Kimscope HeadLight^TM^ SLL-05, Kimscope, Seoul, Korea). The Intraclass correlation coefficient, ICC is 0.89.

Prior to the oral examinations, the Hapcheon Public Health Center and Hapcheon Education Support Office gave their approval for conducting oral examinations on children and adolescents attending school, and the oral examinations were conducted at the school site in June and July 2015. Results from the oral examinations conducted by a dentist were directly recorded into a specially designed datasheet by trained assistants. 

### 2.3. Statistical Analysis

The results of the oral examinations were analyzed using IBM SPSS Statistics 23.0^®^ (IBM Corp. Chicago, IL, USA). The confounding parameters such as the gender and number of sealant teeth were adjusted to compare the DMFT scores recorded pre-CWFP in 2000 and post-CWFP in 2015 for the estimation of the caries-reducing effect derived in 15 years. The mean number of sealant teeth and the gender of the participants in each age group were adjusted to confirm that there were no differences in the oral health status observed between the Hapcheon and control groups before the CWFP was implemented in 2000. On the other hand, by comparing the DMFT scores among children and adolescents in Hapcheon in 2015 to the national data from the 6th KNHANES (2013–2015), another set of data representing the caries-reducing effect derived from the 15-year implementation of CWFP were estimated. The total population of Korea estimated by the National Statistics Korea in 2015 was 51,069,375 [23], and the population under the CWFP nationwide is 2,394,530 [11]. The CWFP beneficiary population was only 4.7% of the total Korean population, and the national data pertaining to each age group could be used as control data. The significance of the differences between gender groups was determined using the chi-square test. The differences between the DMFT scores of the two groups were examined by an independent samples t-test. To adjust for the effects of confounding variables owing to the differences in the number of sealants between the control and CWFP groups, a covariance analysis (ANCOVA) and logistic regression analysis were used. A difference was considered to be statistically significant at *p* < 0.05. 

## 3. Results

Per the results of the survey conducted before the CWFP implementation in 2000, there was a larger proportion of girls in the control group and a larger proportion of boys in the Hapcheon group (*p* = 0.048). There was a significant difference in gender distribution between the control and Hapcheon groups among the 8-year-olds (*p* = 0.007). However, the genders were similarly distributed among 10- and 12-year-old groups in both the control and Hapcheon groups (Table 1). 

Per the results of the survey conducted 15 years post-CWFP in 2015, the gender distribution between the control (KNHANES in 2013–2015) and CWFP groups was not significantly different among all the age groups (Table 1). There were no significant differences in the gender distribution between the Hapcheon sample in 2000 and the sample in 2015 across the four age groups (not shown in table).

The mean number of sealant teeth refers to the mean value of the number of permanent teeth that received sealant treatment among the subjects. The mean number of sealant teeth recorded in 2000 was higher in Hapcheon (2.24) than in the control group from the national data (0.47) (*p* < 0.001). Concerning the distribution by age, among the 8- and 10-year-olds the number of sealant teeth was higher in Hapcheon than in the control group; however, among the 12-year-olds, there were no significant differences observed between the two groups (*p* = 0.397). Per the results of the survey conducted 15 years post-CWFP, the mean number of sealant teeth was not significantly different between the control and Hapcheon groups (*p* = 0.077). There were no significant differences in the number of sealant teeth between the 8- and 15-year-old groups. However, among the 10- and 12-year-olds, the number of sealant teeth were higher in the control group than in Hapcheon. (*p* < 0.05) (Table 2). 

Table 3 shows the adjusted DMFT scores and caries-reducing effects, which were estimated using an analysis of covariance (ANCOVA) after adjusting for age, gender, and the number of sealant teeth. The DMFT score is the average of the sum of the number of decayed teeth, missing teeth due to dental caries, and teeth filled by dental treatment among the permanent dentition. The adjusted DMFT scores were not significantly different between the control and Hapcheon groups in 2000 when the CWFP was introduced in Hapcheon. 

In 2015, there were significant differences observed in the adjusted DMFT scores between the control and Hapcheon groups. In relation to the age groups, among the 8-year-olds, there were no significant differences between the two groups; however, among the 10-, 12- and 15-year-olds, the adjusted DMFT scores recorded in Hapcheon were less than those of the control group (*p* < 0.05) (Table 3). In order to assess the caries-reducing and preventive effects following 15 years of the CWFP implementation, a cross sectional analysis was conducted between the control (national data collected between 2013–2015) and Hapcheon groups in 2015. The overall DMFT scores were lower in Hapcheon than in the control group; further, the caries-preventive effects were evaluated as follows: overall: 29.6%, at age 10 years: 43.2%, at age 12 years: 37.0%, and at age 15 years: 25.5% (Table 3).

**Figure 2:**
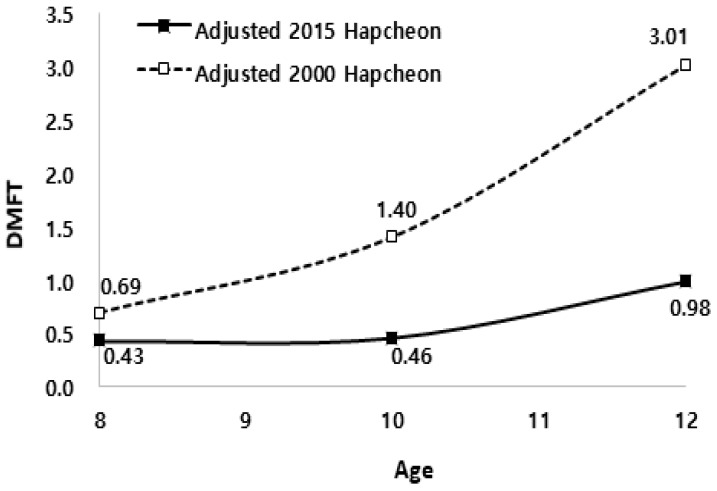
DMFT scores adjusted for gender and the number of sealant teeth recorded in 2000 and 2015, Hapcheon township area.

The results of the evaluation of the caries-reducing effects derived between 2000 and 2015 (15 years) in Hapcheon are as follows: among the 8-, 10-, and 12-year-olds, the adjusted DMFT scores recorded in 2015 were lower than those recorded in 2000 (*p* < 0.05); further, the overall caries-reducing effect was 66.9%, and by age it was as follows: at age 8: 37.7%, at age 10: 67.1%, and at age 12: 67.4% (*p* < 0.05) (Table 4, Figure 2). 

## 4. Discussion

The purpose of this study was to evaluate the caries-reducing effects of the CWFP that was implemented for 15 years from 2000 in the suburban Hapcheon township area, Korea. This study is a 15-year follow-up study that compares the oral health data of children and adolescents recorded at the beginning of the CWFP in 2000 with those recorded in 2015 in Hapcheon; further, it is concurrently a cross-sectional study that compares the oral health data of children and adolescents recorded in 2015 in Hapcheon with the national data collected from the KHANES, 2013–2015. The both caries-reducing effect evaluated in cross-sectional comparative study and the 15-year follow-up study were confirmed the caries reducing effect of CWFP in suburban area.

The interventions that can effectively reduce the incidence of dental caries are sealants as well as the CWFP [17]. Therefore, it is necessary to adjust for the number of sealant teeth while estimating the dental caries-reducing effects of the CWFP alone in cases where the effects of being treated with sealants overlap with those derived from the CWFP. In addition, there are many cases that have been reported in which the prevalence of dental caries in women is generally higher than in men in the absence of special dental caries preventive interventions. Because, both biological (genetics, hormones, and reproductive history) and anthropological (behavioral) factors such culture-based division of labor and gender-based dietary preferences play a role in the incidence of dental caries. [24]. Thus, when the gender distributions within the sample groups are found to be different, it is necessary to adjust for the difference in gender distribution. Per the results of the surveys conducted on the control and Hapcheon groups in 2000, there were significant differences in gender distribution among the groups included as the control and Hapcheon groups.

In 2000, among the 12-year-olds, the number of sealant teeth in the Hapcheon group was not significantly different from in the control group (national survey); however, among the 8- and 10-year-olds, the number of sealant teeth in Hapcheon was higher. These results were evaluated owing to the sealant program conducted, since 1996, by the Hapcheon Public Health Center that involved visiting primary schools [17]. Comparing the number of sealant teeth in the control group recorded between 2013–2015 to in the Hapcheon township area recorded in 2015, the number of sealant teeth was lower in the Hapcheon township area compared to in the control group (whole country). Fifteen years following the CWFP implementation, in 2015, the numbers of sealant teeth in Hapcheon was less than it was in 2000. This means that the number of sealant teeth in Hapcheon has decreased compared to the national average after the 15-year period in which the CWFP was implemented. Since 2010, the Korea Ministry of Health and Welfare has cancelled the supplementary budget for the sealant program conducted by the community public health center, in which children themselves had to visit the community public health centers or private dental clinics for the application of sealants that were provided by the national health insurance. With the benefits provided by the national health insurance for sealant application, although the free sealant program that involved school visits by the Hapcheon Public Health Center stopped, the copayment charge of 15-30% was provided depending on a subject's family income. However, it can be interpreted that the benefits to children who could not visit dental clinics or who found it economically burdensome to seek dental treatment have been reduced. Choi et al. [25] reported that the availability of dental sealant treatments was reduced in areas with low access to dental care after the free sealant program was stopped. Therefore, it is necessary to adjust for the difference in gender distribution and sealant status as confounding variables in the evaluation of the caries-reducing effect derived from the 15-year implementation of CWFP in Hapcheon.

Per the results of the cross-sectional comparative study that compared the overall adjusted DMFT score recorded in Hapcheon in 2015 with the overall adjusted DMFT score of the national control group recorded between 2013–2015 via the KNHANES, the caries-reducing effect was estimated to be 29.6% in the total samples. Per the results of the follow-up study that compared the overall adjusted DMFT score recorded in Hapcheon in 2000 with the overall adjusted DMFT score recorded in Hapcheon in 2015, the caries-reducing effect was estimated to be 66.9%. These results confirm that the caries-reducing effect evaluated in the follow-up study was higher. Jeong et al. [26] also reported that the caries-reducing effect was maintained via the CWFP in Hapcheon.

The CWFP in Hapcheon began in 2000 and has been in operation for 15 years. In the case of people who are growing up while drinking tap water containing fluoride at an appropriate concentration since their birth, a large number of fluoroapatite-rich teeth are formed in the crown during the eruption of the permanent teeth, and after a tooth has erupted, the fluoride in the tap water is continuously applied to its surface so that the maximum caries-reducing effect can be expected [27,28]. Singh and Spencer [29] explain the effects of the CWFP in two stages. First, the effect of pre-eruption exposure to fluoride, which involves the consumption of adequate levels of fluoride concentrations in the water before the eruption of teeth, results in teeth that are formed with a large amount of fluoroapatite during the crown-formation period. Second, the post-eruption exposure effect, which occurs after the eruption of the teeth and involves fluoride being applied to the surface of the teeth during the intake of fluoride in drinking water, increases the resistance to caries by increasing the amount of fluoroapatite on the tooth surface. Although these two effects work concurrently, the effect that is primarily involved varies by the area in which the tooth is located. Pre-eruption exposure plays a major role in the reduction of caries on the pit and fissure surfaces; further, the pre-eruption exposure was also effective in reducing the caries on the smooth surfaces; however, the post-eruption exposure also had an influence [29]. If we consider that 95.7% of the DMF surfaces on the permanent dentition of 11-year-old children develop on pit and fissure surfaces in Korea [30], among the various interventions that involve the use of fluoride for caries prevention, the CWFP can be considered as a higher priority intervention that can be applied in Korea. 

Saliva et al. [31] reported that, in Brazil, the DMFT scores decreased by 57.1% by the age of 12 years in all residents that consumed drinking water with appropriate fluoride concentrations after birth. Mahoney et al. [32] reported a 24% reduction in the DMFT scores of more than 50% of people living in less than 10% of the CWFP area during their lifetime. Do et al. [33] and Crocombe et al. [34] reported that the prevalence of DMF surfaces were lower in people who lived a larger proportion of their lives in a CWFP area compared to in people who lived a smaller proportion of their lives in a CWFP area. The caries-reducing effects of the CWFP in Korea have also been reported on several times [15,35]. 

On the other hand, it has been reported that the dental caries prevalence among the socioeconomically challenged population is high and that the oral health among such populations is not in a good condition due to not receiving proper oral care. Song et al. [36] reported that DMFT scores were high among adults with low levels of education or low incomes. Kim et al [37]. reported that the number of lost teeth was high in the elderly with low educational levels.

Further, Kim et al. [38] reported that economic reasons are the most important cause responsible for the lack of dental care among Korean adults. Schwendicke et al. [39] also reviewed literatures on the correlation between socioeconomic factors and dental caries occurrence and concluded that the risk of dental caries increases with a low socioeconomic status. Thus, reducing the incidence of dental caries using the CWFP can help to alleviate the oral health disparities among socially and economically disadvantaged populations. Moreover, Crocombe et al. [40] demonstrated that, among those who spent a large proportion of their lives in a CWFP region, inequalities in oral health conditions due to socioeconomic factors or the lack of dental care were not observed. McGrady et al. [41] reported that the inequalities observed in the prevalence of dental caries owing to socioeconomic levels were alleviated in the UK CWFP region. Among the Australian children from CWFP regions, differences in the incidence of dental caries due to the socioeconomic status was reported to be lower than in the children from areas without the CWFP [42]; the same effect has been observed in New Zealand CWFP regions [43]. It has been reported that, in the US, the CWFP contributes to increasing the uniformity in the social hierarchy [44]. Studies on the inequality in oral health conditions have widely reported that the disparity has been reduced by the CWFP in Korea [45,46]. 

On the other hand, the economic benefits derived from the CWFP have also been reported on by Kim et al. [47] who observed that the benefit-cost ratio of CWFP for 11 years was estimated to be 41.4, which is the ratio estimated by the total benefits derived from the saved costs of caries treatment owing to the CWFP divided by the total cost of the CWFP for 11 years in Jinju, Korea. Cho et al. [48] also analyzed dental health care utilization for 14 years from 2003 to 2013 via data from the Korean National Health Insurance; they compared the data between an area under the CWFP with an area without the CWFP, and reported that the requirements of dental care visits was lower in the CWFP region compared to in the region without the CWFP and that the number of dental visits was lower among individuals who already underwent a dental care visit; further, the duration of the CWFP had an inverse association with the dental care expenditure of the community residents.

Although many positive effects of the CWFP have been reported, some people who do not understand the safety associated with the CWFP have repeatedly suggested that there are risks associated with it, which has led to a decline in the number of areas implementing the CWFP. Despite a number of reports from professors that have majored in preventive medicine demonstrating the safety of CWFP [49], some small groups continue to insist on unproven risks, which causes difficulties in implementing the CWFP. This study, which reports on the long-term caries-reducing effects derived from the CWFP, can contribute to the evidence on the benefits gained from it. Further researches on this subject will be needed in the future. 

Although there are lots of changes, such as in socioeconomic levels and diets, that may affect the incidence of dental caries over a long period of more than 10 years, in this study, we did not include these variables in the survey due to limitations concerning privacy and research resources. In addition, children born in other places who migrated to Hapcheon were not excluded from the survey. The detailed effect of CWFP can be demonstrated by including data related to socioeconomic status, household usage of tap water, and oral health behaviors of individuals. Despite these limitations, this study could confirm the caries-reducing effect of a 15-year CWFP implemented in the Hapcheon township area, and our data fully support the feasibility of the CWFP.

## 5. Conclusions

The both caries-reducing effect evaluated in cross-sectional comparative study and the 15 years follow-up study were confirmed the caries reducing effect of CWFP in suburban area. The effect of CWFP underwent sealant teeth together, which are two priorities considered to reduce dental caries in children and adolescents, was confirmed. These results suggest that the its effect from CWFP in suburban Hapcheon township area was so high that the fluoridation program should be developed in other regions in Korea as well. Health policy makers should consider CWFP as a priority policy for caries-reducing in Korean children and adolescents.

## Figures and Tables

**Table 1:** Distribution of subjects by age and gender.

**Before CWFP**	Control (National Data in 2000) *			Hapcheon in 2000 **			***p*** **-Value ^‡^**
	Total (Gender)	Boy	Girl	Total (Gender)	Boy	Girl	
Total (Age)	3602	1797	1805	671	363	308	0.048
8	1194	592	602	213	127	86	0.007
10	1205	600	605	198	98	100	0.939
12	1203	604	599	260	138	122	0.412
**After 15 Years of CWFP**	**Control (National Oral Health Data in 2013–2015) ^†^**			**Hapcheon in 2015**			***p*** **-Value ^‡^**
	Total (Gender)	Boy	Girl	Total (Gender)	Boy	Girl	
Total (Age)	952	519	433	498	174	162	0.698
8	243	122	121	103	53	50	0.906
10	239	133	106	116	58	58	0.364
12	239	130	109	117	63	54	1.000
15	231	134	97	162	92	70	0.836

* Participants in the Korean National Oral Health Survey in 2000 as the control group. ** Results from oral examinations conducted before the implementation of the CWFP. ^†^ Participants in the 6th KNHANES conducted in 2013–2015 as the control group. ^‡^ Chi-square test conducted on the gender distribution between the Hapcheon and control groups before and 15 years after the CWFP implementation, respectively. CWFP: Community water fluoridation program. KNHANES: Korea National Health and Nutrition Examination Survey. The Hapcheon township area had benefited from the CWFP from 2000 to 2015.

**Table 2:** Mean numbers of sealant teeth.

**Before CWFP**	Control (National Data in 2000) *			Hapcheon in 2000 **			***p*** **-Value** **^‡^**
	*N*	Mean	SE ^‡^	*N*	Mean	SE ^‡^	
Total (Age)	3602	0.47	0.02	671	2.24	0.10	<0.001
8	1194	0.49	0.03	213	1.90	0.08	<0.001
10	1205	0.48	0.04	198	5.06	0.19	<0.001
12	1203	0.44	0.04	260	0.36	0.07	0.397
**After 15 Years of CWFP**	**Control (National Data in 2013–2015) ^†^**			**Hapcheon in 2015**			***p*** **-Value** **^‡^**
	*N*	Mean	SE ^‡^	*N*	Mean	SE ^‡^	
Total (Age)	952	1.67	0.07	498	1.48	0.08	0.077
8	243	1.62	0.11	103	1.61	0.15	0.959
10	239	1.76	0.11	116	1.26	0.13	0.005
12	239	1.85	0.16	117	1.14	0.14	0.001
15	231	1.42	0.18	162	1.80	0.17	0.149

* Participants in the Korean National Oral Health Survey conducted in 2000 as the control group. ** Results from oral examinations conducted before the implementation of CWFP. ^†^ Participants in the 6th KNHANES conducted between 2013–2015 as the control group. ^‡^ Independent samples t-test results between Hapcheon and control groups comparing the mean numbers recorded pre-CWFP and 15 years post-CWFP, respectively. CWFP: Community water fluoridation program. KNHANES: Korean National Health and Nutrition Examination Survey. The Hapcheon township area was under the CWFP from 2000 to 2015.

**Table 3:** Adjusted DMFT scores of the control and Hapcheon groups before and after the CWFP implementation and the caries-reducing effects assessed by a comparison between the adjusted DMFT scores.

**Before CWFP in 2000**	Control in 2000 *			Hapcheon in 2000 **				***p*** **-Value**
	*N*	Mean	SE ^‡^	*N*	Mean	SE ^‡^		
Total (Age)	3602	1.82 ^‡^	0.04	671	1.98 ^‡^	0.09	-	0.094 ^‡^
8	1194	1.00 ^§^	0.04	213	0.92 ^§^	0.10	-	0.456 ^§^
10	1205	1.59 ^§^	0.06	198	1.75 ^§^	0.18	-	0.402 ^§^
12	1203	2.86 ^§^	0.08	260	3.04 ^§^	0.17	-	0.346 ^§^
**After CWFP for 15 years**	**Control (National Data in 2013–2015) ^†^**			**Hapcheon in 2015**			**Caries Reducing Effects**	***p*** **-Value**
	*N*	Mean	SE ^‡^	*N*	Mean	SE ^‡^	%	
Total (Age)	952	1.42 ^‡^	0.06	498	1.00 ^‡^	0.09	29.6	<0.001 ^‡^
8	243	0.44 ^§^	0.06	103	0.50 ^§^	0.10	-	0.615 ^§^
10	239	0.88 ^§^	0.08	116	0.50 ^§^	0.12	43.2	0.009 ^§^
12	239	1.38 ^§^	0.12	117	0.87 ^§^	0.17	37.0	0.017 ^§^
15	231	2.82 ^§^	0.20	162	2.10 ^§^	0.24	25.5	0.019 ^§^

Caries reducing effects = [(Control − Hapcheon in 2015)/Control]) × 100. * Participants in the national oral health survey conducted in 2000. ** Results from oral examinations conducted before the implementation of the CWFP. ^†^ Participants in the KNHANES conducted in 2013–2015. ^‡^ Results from ANCOVA adjusted for age, gender, and number of dental sealants. ^§^ Results from ANCOVA adjusted for gender and number of dental sealants. CWFP: Community water fluoridation program. KNHANES: Korea National Health and Nutrition Examination Survey. The Hapcheon township area is a CWFP area.

**Table 4:** Caries-reducing effects derived from a 15-year CWFP implementation using the adjusted DMFT scores in Hapcheon.

Age	Hapcheon in 2000 *			Hapcheon in 2015			Caries Reducing Effects (%)	*p*-Value
	*N*	Mean	SE ^†^	*N*	Mean	SE ^†^		
Total	671	1.81 ^‡^	0.07	336	0.60 ^‡^	0.10	66.9	<0.001 ^‡^
8	213	0.69 ^§^	0.08	103	0.43 ^§^	0.11	37.7	0.001 ^§^
10	198	1.40 ^§^	0.11	116	0.46 ^§^	0.16	67.1	0.008 ^§^
12	260	3.01 ^§^	0.15	117	0.98 ^§^	0.22	67.4	<0.001 ^§^

Caries reducing effects = [(Hapcheon in 2000 − Hapcheon in 2015)/Hapcheon in 2000]) × 100. * Results from oral examinations before the implementation of the CWFP in Hapcheon. ^†^ Standard error. ^‡^ Results from ANCOVA adjusted for age, gender, and the number of sealant teeth. ^§^ Results from ANCOVA adjusted for gender and the number of sealant teeth.
